# Deep Learning–Based Estimation of Radiographic Position to Automatically Set Up the X-Ray Prime Factors

**DOI:** 10.1007/s10278-024-01256-x

**Published:** 2024-10-14

**Authors:** C. F. Del Cerro, R. C. Giménez, J. García-Blas, K. Sosenko, J. M. Ortega, M. Desco, M. Abella

**Affiliations:** 1 Dept. Bioingeniería, Universidad Carlos III de Madrid, Leganés, Madrid, Spain; 2 Instituto de Investigación Sanitaria Gregorio Marañón, Madrid, Spain; 3 Dept. de Informática, Universidad Carlos III de Madrid, Madrid, Spain; 4 Sociedad Española de Electromedicina y Calidad S.A. (SEDECAL), Madrid, Spain; 5 Centro Nacional de Investigaciones Cardiovasculares Carlos III (CNIC), Madrid, Spain; 6 Centro de Investigación en Red en Salud Mental (CIBERSAM), Madrid, Spain

**Keywords:** Radiography, Radiographic position, Prime factors, Deep learning, Classification

## Abstract

Radiation dose and image quality in radiology are influenced by the X-ray prime factors: KVp, mAs, and source-detector distance. These parameters are set by the X-ray technician prior to the acquisition considering the radiographic position. A wrong setting of these parameters may result in exposure errors, forcing the test to be repeated with the increase of the radiation dose delivered to the patient. This work presents a novel approach based on deep learning that automatically estimates the radiographic position from a photograph captured prior to X-ray exposure, which can then be used to select the optimal prime factors. We created a database using 66 radiographic positions commonly used in clinical settings, prospectively obtained during 2022 from 75 volunteers in two different X-ray facilities. The architecture for radiographic position classification was a lightweight version of *ConvNeXt* trained with fine-tuning, discriminative learning rates, and a one-cycle policy scheduler. Our resulting model achieved an accuracy of 93.17% for radiographic position classification and increased to 95.58% when considering the correct selection of prime factors, since half of the errors involved positions with the same KVp and mAs values. Most errors occurred for radiographic positions with similar patient pose in the photograph. Results suggest the feasibility of the method to facilitate the acquisition workflow reducing the occurrence of exposure errors while preventing unnecessary radiation dose delivered to patients.

## Introduction

The quality of a radiograph, i.e., contrast resolution and signal-to-noise ratio, depends on the optimal tuning of the so-called prime factors: source voltage (KVp), source current and exposure time (mAs), and distance from the source to the detector (SDD). These factors that depend on the radiographic position are typically adjusted by a technician prior to acquisition using pre-calculated values based on standards [1] and guidelines from the system manufacturer. However, a wrong selection of these factors can result in non-diagnostic images, requiring repetition and exposing the patient to additional ionizing radiation. In fact, studies have shown that the repeat/rejection ratio in clinical practice is between 13% and 14% [2]. While most modern systems have an *Automatic Exposure Control* (AEC) that interrupts the radiation when the exposure is sufficient at the detector [3], it is not effective when prime factors are smaller than necessary. Therefore, an automatic mechanism to adjust prime factors to optimal values depending on the patient pose would be highly beneficial.

To the best of our knowledge, no other work has been published that addresses this problem. Several studies have explored the use of *convolutional neural networks* (CNN) to automatically detect radiographic positions in the acquired radiography for quality control [4, 5]. Other studies have used pretrained networks such as *VGG* [6] or *ResNet* [7] to classify chest radiographs into anteroposterior or posteroanterior views [8, 9], or to detect the radiographic position of hands [10]. However, these methods rely on acquired X-ray images for position detection, which does not prevent the patient from receiving an extra radiation dose in case of errors.

On the other hand, the identification of human body parts in photographs has been done with *You Only Look Once* (*YOLO*) [11], but this model is not able to distinguish between different human poses [12]. Other works described the human pose by a complete representation using key points [13], in a variety of clinical applications: estimating the position of clinicians for surgical workflow analysis [14, 15] or in-bed patients for monitoring [16], and analyzing infant motion for early detection of cerebral palsy [17]. Nevertheless, an accurate estimation of key points involves manual annotation, which is time-consuming and not necessary for the classification problem of radiographic position estimation. In [18], the authors optimized a transfer learning methodology for a CNN to classify human activities, but the application of this approach for radiographic position estimation in a clinical setting is yet to be evaluated. In this work, we intend to fill this gap by developing an automatic method for the detection of the radiographic position of the patient to set up the X-ray prime factors. Our main contributions include the creation of an extensive database containing photographs of patients in a clinical setting for the most common radiographic positions used in clinical practice. We combined transfer learning with a cutting-edge deep-learning architecture to develop a model intended for real-time applications that uses a photograph of the patient positioned in the system to estimate the radiographic position.

## Material and Methods

The proposed method estimates the radiographic position from a photograph of a patient. This information is used to determine the parameters of the X-ray source (KVp and mAs) by looking up a predefined table to ensure image quality. The following sections describe the database, the resulting model, the evaluation methodology, and the creation of an executable program to be used in a real system.

### Database

We selected 66 radiographic positions commonly used in clinical settings (see Table 1), from 75 volunteers. These positions were chosen in collaboration with radiology technicians from Gregorio Marañón University Hospital (Madrid, Spain). Data acquisition was conducted in two locations, one for 50 volunteers and a different one for the remaining 25 volunteers. Although no radiation was involved in the acquisition, all participants signed a legal consent that recognizes the protection of their data as established in national and international regulations (General Data Protection Regulation, EU Regulation 2016/679).

**Table 1:** List of the 66 radiographic positions used in the study, categorized by body region

Body region	Positions
Head	Skull PA, Skull LAT, Sinus, Nasal Bones LAT
Spine	Cervical Spine AP, Cervical Spine LAT, Thoracic Spine AP, Thoracic Spine LAT, Lumbar Spine AP, Lumbar Spine LAT
Shoulder	Shoulder Left AP, Shoulder Left Axial, Shoulder Right AP, Shoulder Right Axial
Upper limb	Elbow Left AP, Elbow Left LAT, Elbow Right AP, Elbow Right LAT, Wrist Left AP, Wrist Left LAT, Wrist Right AP, Wrist Right LAT, Hand Left AP, Hand Left OBL, Hand Right AP, Hand Right OBL, Fingers Left 1st AP, Fingers Left 1st LAT, Fingers Left 3rd AP, Fingers Left 3rd LAT, Fingers Right 1st AP, Fingers Right 1st LAT, Fingers Right 3rd AP, Fingers Right 3rd LAT
Torso	Thorax PA, Thorax LAT, Abdomen AP, Ribs Left AP, Ribs Right AP, Pelvis AP, Hip Left AP, Hip Left Axial, Hip Right AP, Hip Right Axial, Sacro-coccyx AP, Sacro-coccyx LAT
Lower limb	Femur Left AP, Femur Left LAT, Femur Right AP, Femur Right LAT, Knee Left AP, Knee Left LAT, Knee Right AP, Knee Right LAT, Tibia Left AP, Tibia Left LAT, Tibia Right AP, Tibia Right LAT, Ankle Left AP, Ankle Left LAT, Ankle Right AP, Ankle Right LAT, Foot Left AP, Foot Left OBL, Foot Right AP, Foot Right OBL

**Fig. 1:**
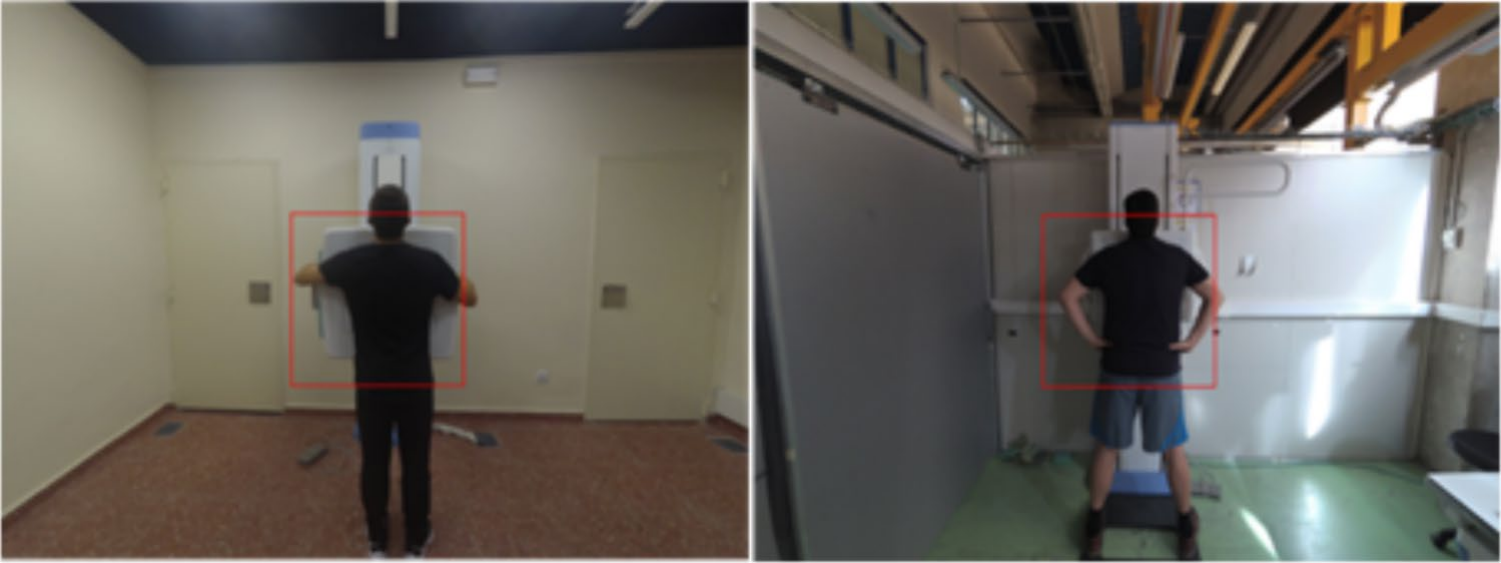
Example of thorax AP position at both facilities

The data acquisition setup in both facilities consisted of an X-ray table, a wall stand, and a ceiling-mounted X-ray source equipped with a YI 4 K Action camera attached to the collimator case using a monopod. To minimize the influence of background elements, we cropped the photographs to a square region with a width of 90 cm, corresponding to the width of the X-ray table (red square in Fig. 1), considering the 19 cm offset on the vertical axis of the camera with respect to the collimator light.

**Fig. 2:**
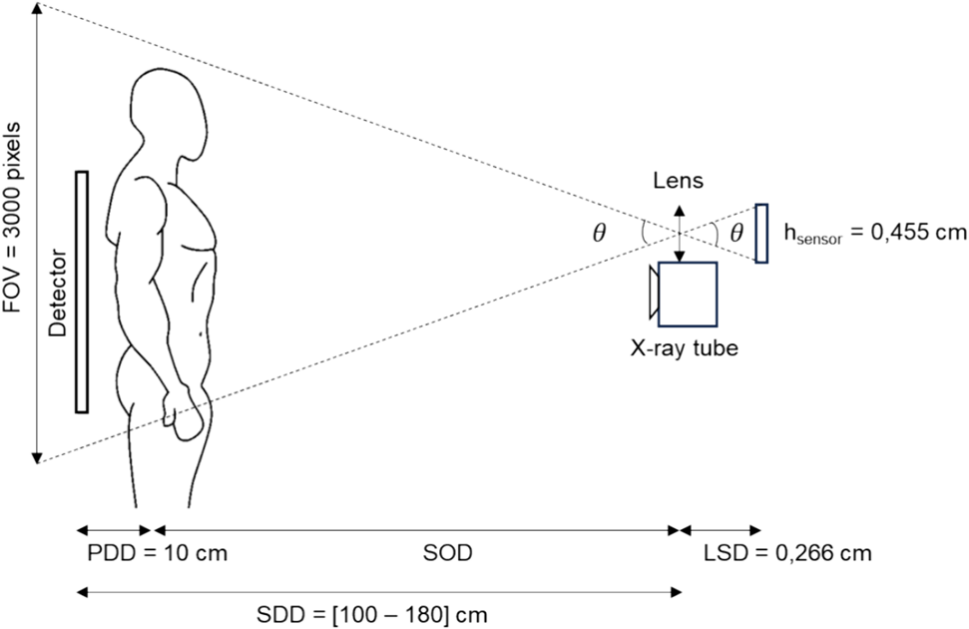
Ray-tracing diagram of the FOV with respect to the camera lens and sensor size

The pixel size to calculate the cropped area was determined using Eq. 1, as illustrated in Fig. 2.

1 $$PixSize={FOV}_{mm}/{FOV}_{px}=\left({h}_{s}\times {SOD}_{cm}/{LSD}_{cm}\right)/{FOV}_{px}=m\times {SOD}_{cm}=m\times \left({SDD}_{cm}-{PDD}_{cm}\right)$$

where ${h}_{s}$ is the height of the camera sensor, ${LSD}_{cm}$ is the distance between the lens and camera sensor, ${FOV}_{px}$ is the field of view in pixels, $m$ is a camera-dependent slope, ${SDD}_{cm}$ is the distance between the source and detector, and ${PDD}_{cm}$ is the distance between the patient and detector (10 cm in our case). The calculated pixel size was 0.0513 cm, 0.0798 cm, and 0.0969 cm for SDD of 100, 150, and 180, respectively.

### Proposed Model

**Fig. 3:**
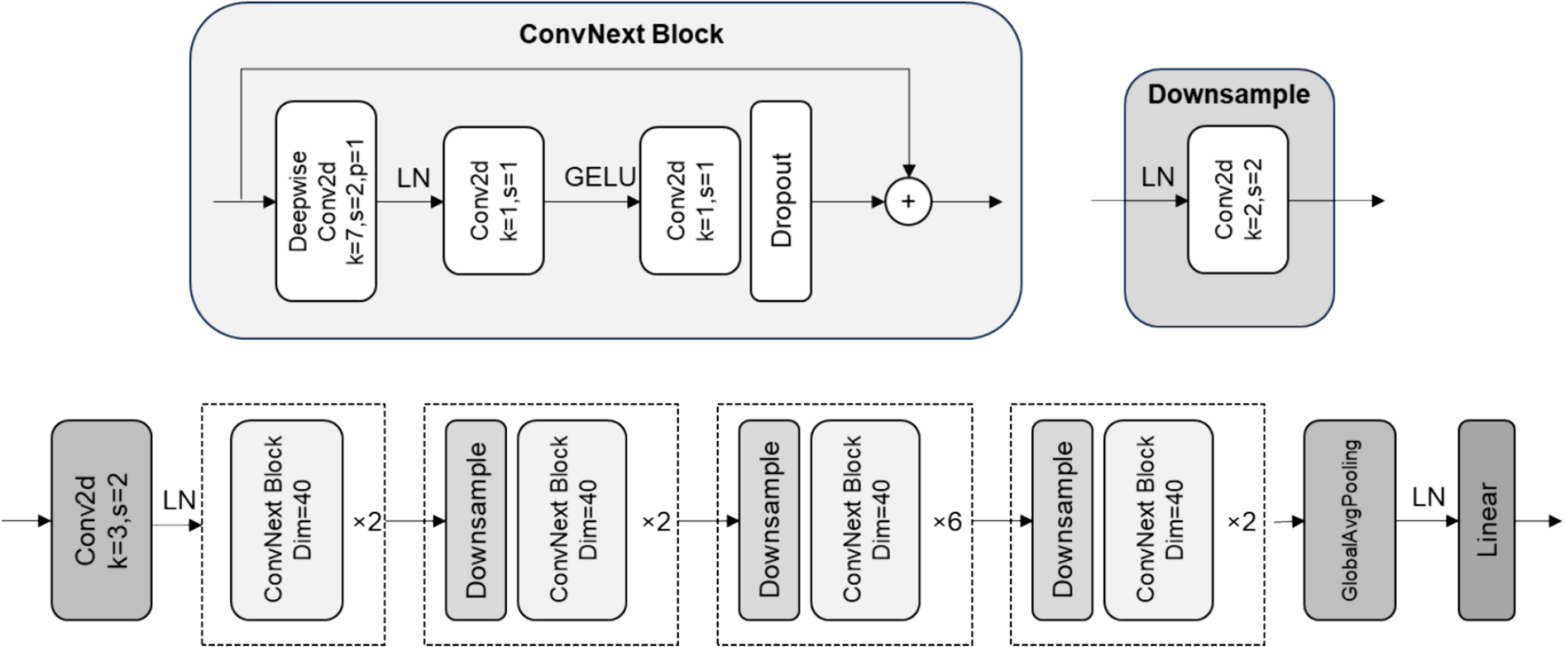
Proposed architecture. Conv2d, convolution layers; LN, normalization layer; k, kernel size; s, stride; and p, padding

We adapted the lightweight *atto* version of the *ConvNeXt* architecture (CN) [19] from *Pytorch Image Models* (timm) [20], to accommodate the 66 radiographic positions considered in our study and enable real-time inference (Fig. 3). The loss function was cross-entropy, and we used the Ranger optimizer [21].

We conducted three experiments, resulting in a total of seven models. In the first experiment, we compared the performance of using color and grayscale images to determine the optimal color mode. In the second experiment, we used the selected color mode and evaluated the impact of three types of data augmentation and their combinations on the training set. This doubled the size of the database for one augmentation and increased it fourfold when combining all the augmentations. Data augmentations were horizontal flip, random zoom with a scale factor between 1.1 and 1.3, and random rotation of an angle θ drawn from a Gaussian distribution with a mean of 0 and a standard deviation of 10 degrees. We trained end-to-end these six pretrained models pretrained with *ImageNet-1K* [22] and obtained an optimal learning rate of 1 × 10^−3^ with the Leslie N. Smith test [23]. In the third experiment, we added fine-tuning (FT), discriminative learning rates (DLR) [24], and a one-cycle policy (OCP) scheduler [23]. The Leslie N. Smith indicated an optimal learning rate of 1 × 10^−3^ when all layers were frozen except for the last one. When training the model end-to-end, we used discriminative learning rates of 1 × 10^−6^, 1 × 10^−5^, and 1 × 10^−4^ for the first, second, and last third of our model, respectively, as determined by the same test.

Input images were rescaled to 224 × 224 pixels and normalized based on the mean and standard deviation of *ImageNet-1K*.

### Implementation

**Fig. 4:**
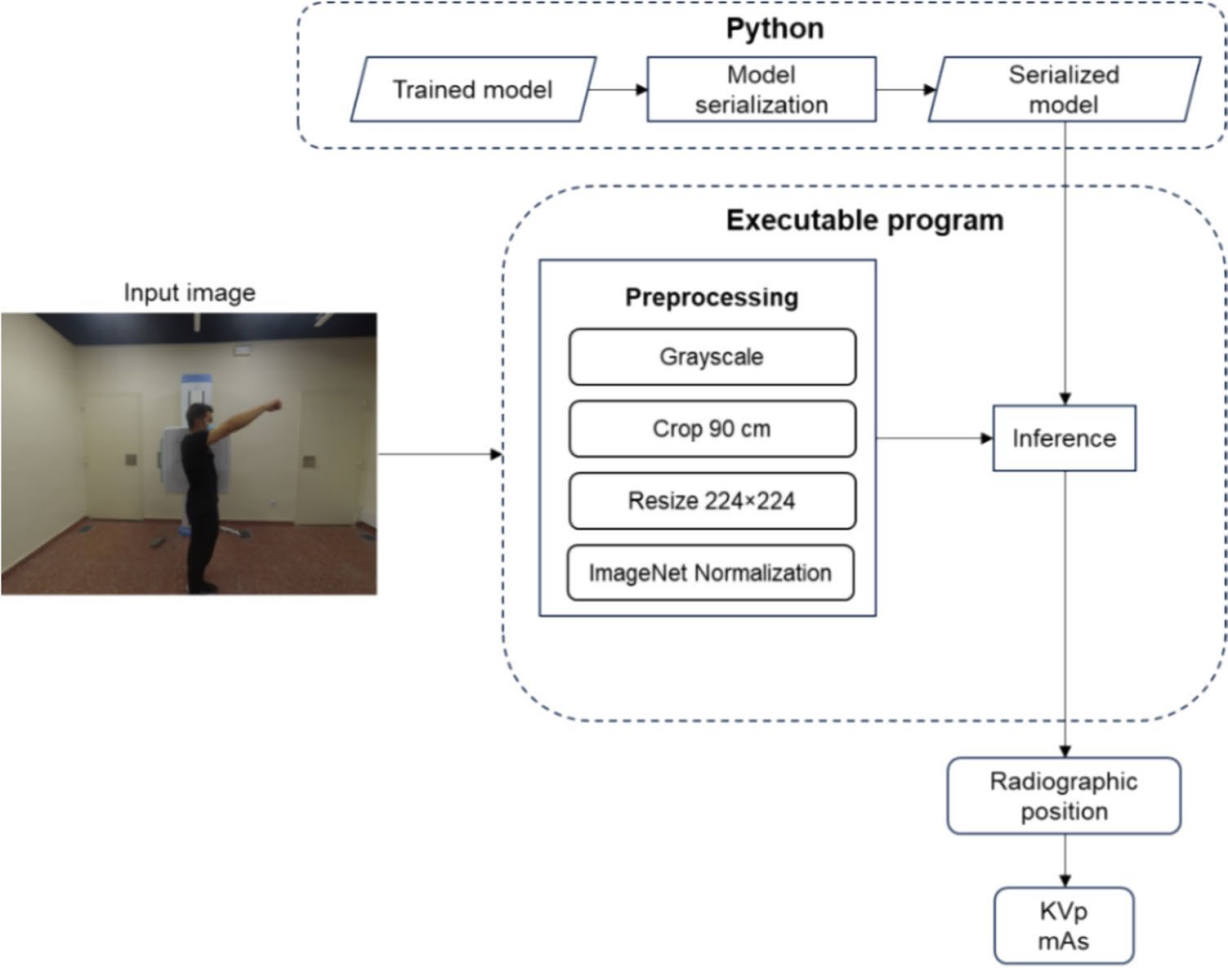
Workflow of the conversion from trained models to the final executable

The proposed model was implemented in* Python* [20], using *PyTorch* [25] and *Fastai* [26] libraries. We then developed an executable program using *LibTorch*, a C ++ library that provides an API for *PyTorch*, and *OpenCV* [27] for image preprocessing. To ensure compatibility between *PyTorch* and *LibTorch*, we used *Torch Script*. The program was compiled for Windows® machines with × 86–64 processor architecture and can perform image inference using only the CPU, without requiring *Python* installation. The workflow involves converting the photograph to grayscale, cropping it to a 90-cm square region, resizing it to 224 × 224 pixels, and normalizing it based on the mean and standard deviation of *ImageNet-1 K* (Fig. 4). This preprocessed image is then passed through our model to infer the radiographic position, which is used to automatically determine the appropriate KVp and mAs values based on a predefined table.

### Evaluation Methodology

To verify the robustness of the models given the limited size of the database, we performed 5-fold cross-validation with five randomly chosen validation sets for all experiments, splitting the dataset into 60 volunteers for training and 15 for validation.

Since some radiographic positions share the same prime factors, we evaluated the accuracy of the proposed models in terms of both radiographic position identification and prime factor values. To assess classification errors, we used confusion matrices generated from the validation sets.

We compared our architecture with two lightweight state-of-the-art models with a similar number of parameters (3.7M), EfficientFormerV2 (EF) [28] and MobileNetV4 (MN) [29], both from the *timm* library. The chi-square test was used to assess differences in the accuracy rates for each model.

## Results

Table 2 presents the mean and standard deviation of the 5-fold cross-validation results for each architecture, considering both grayscale (GS) and color (RGB) models. Our model, CN, achieves superior performance (*p* < 0.05) compared to EF and MN in both radiographic position identification and prime factors accuracy. While the performance using GS and RGB models was very similar, with no significant differences between them, GS showed a shorter inference time (using only one channel).

**Table 2:** Results obtained for each color mode with three architectures. The best result is highlighted in bold, and the second-best result is underlined. *p < 0.05 with respect to CN (GS)

Model	Time/epoch (s) ↓	Best epoch↓	Cross-entropy↓	Position acc (%)↑	Prime factors acc. (%)↑
CN (GS)	12	7 ± 1	**0.27 ± 0.03**	**91.15 ± 1.06**	94.10 ± 0.93
CN (RGB)	14	7 ± 1	0.27 ± 0.04	91.15 ± 1.28	**94.57 ± 1.34**
EF (GS)	12	**6 ± 0**	0.41 ± 0.03	86.48 ± 1.24*	92.01 ± 1.24*
EF (RGB)	14	15 ± 18	0.37 ± 0.03	88.77 ± 1.47*	93.48 ± 0.97*
MN (GS)	**8**	26 ± 9	0.46 ± 0.03	87.90 ± 1.20*	91.51 ± 1.15*
MN (RGB)	10	22 ± 12	0.43 ± 0.02	87.82 ± 0.95*	91.62 ± 0.76*

Table 3 presents the mean and standard deviation of the 5-fold cross-validation results for different data augmentations and training policies for the proposed architecture, CN. The use of individual data augmentations or the combination of all of them did not significantly improve the accuracy of the grayscale model. However, the addition of fine-tuning, discriminative learning rates, and OCP reduced cross-entropy and improved both radiographic position and prime factors accuracy by 2.21% and 1.57% respectively (*p* < 0.05).

**Table 3:** Results obtained for the grayscale models with the proposed architecture CN(GS), adding different data augmentations and training policies. The best result is highlighted in bold, and the second-best result is underlined. *p < 0.05 with respect to CN (GS)

Model	Time/epoch (s) ↓	Best epoch↓	Cross-entropy↓	Position acc (%)↑	Prime factors acc. (%)↑
CN (GS + flip)	**24**	5 ± 1	0.27 ± 0.02	91.00 ± 0.41	93.85 ± 0.88
CN (GS + zoom)	**24**	4 ± 1	0.27 ± 0.03	90.98 ± 0.74	94.22 ± 0.98
CN (GS + rotation)	**24**	5 ± 1	0.27 ± 0.02	91.66 ± 1.00	94.49 ± 1.05
CN (GS + flip + zoom + rotation)	46	**3 ± 2**	0.27 ± 0.01	91.77 ± 0.88	94.54 ± 0.39
CN (GS + flip + zoom + rotation (FT + DLR + OCP))	46	5 ± 0	**0.21 ± 0.02**	93.17 ± 0.80*	95.58 ± 0.82*

**Fig. 5:**
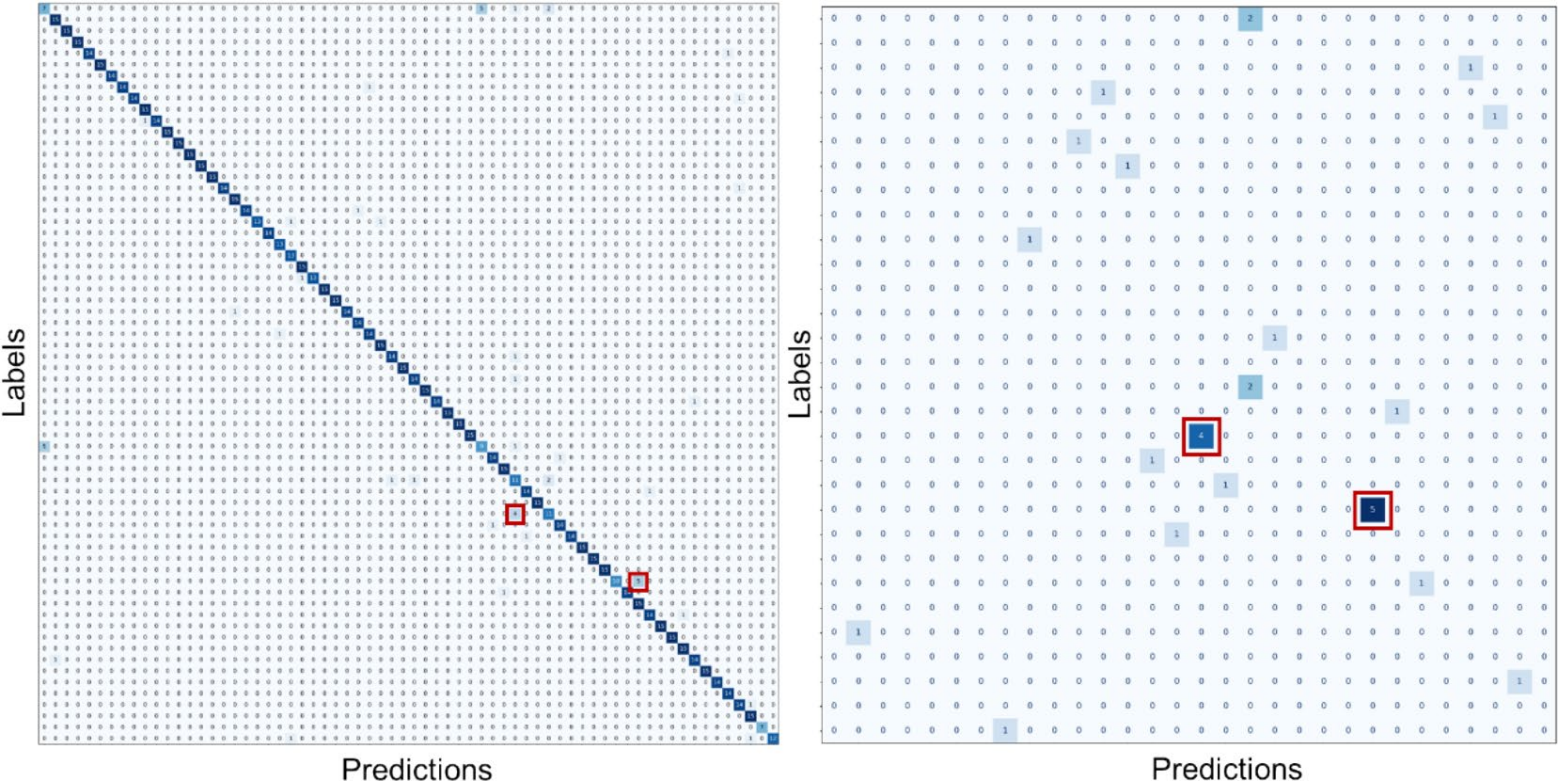
Confusion matrix (left) and errors with different prime factors (right) for one of the validation sets

The left panel of Fig. 5 displays the confusion matrix, with most predictions falling along the diagonal, indicating correct classification. The right panel of Fig. 5 shows that errors in prime factors correspond to similar radiographic positions, such as Skull PA and Sinus (five errors) or Sacro-coccyx AP and Pelvis AP (four errors), which are marked with a red square.

**Fig. 6:**
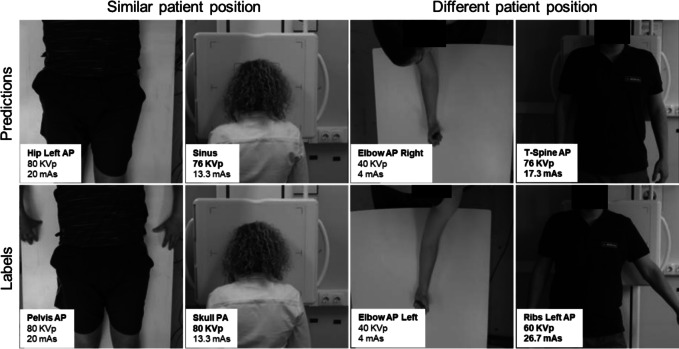
Scenarios with similar (left) and different (right) patient poses. Errors are highlighted in bold

Errors in radiographic position classification occurred in 6% of validation set cases. These errors can be categorized into four classes: similar patient pose with the same prime factors (35%), similar patient pose with different prime factors (46%), different patient poses with the same prime factors (4%), and different patient poses with different prime factors (15%). Fig. 6 provides an example of each class.

## Discussion and Conclusion

We have presented a novel method that estimates the radiographic position from photographs to automatically select the prime factors in real time for X-ray acquisition. To achieve this, we created a database containing photographs of patients in a clinical setting, covering the most common radiographic positions. These photographs were taken with a camera attached to the collimator case, preprocessed, and fed into *ConvNeXt*. This architecture was chosen for its superior balance between efficiency and accuracy, making it well suited for real-time applications.

The comparison of the proposed architecture with previously proposed architectures EfficientFormerV2 and MobileNetV4 showed statistically significant better results. The combination of data augmentation techniques and the application of training policies resulted in statistically significant improvements in performance, achieving an accuracy of 93.17% for radiographic position classification, despite the limited size of our database. Most errors occurred for positions with a similar patient pose in the photograph. The accuracy increased to 95.88% when evaluating the correct selection of prime factors, as half of the errors involved positions with the same KVp and mAs values. Only 1% of the total images corresponded to errors with different patient poses and different prime factors. These errors may be attributed to inconsistencies in our database, as some radiographic positions were acquired at different distances in the two facilities. This problem could be minimized by increasing the database with more examples of these radiographic positions. Nevertheless, the remaining errors would not represent a major issue in a real scenario, as the acquisition would still be supervised by an X-ray technician.

Since the prime factors vary slightly depending on the anatomy of the patient, future research will evaluate the use of 3D cameras to complement the pose information with patient thickness data, estimated from the distance between the patient and the camera sensor. This will involve evaluating changes in the prime factors according to the individual morphology of the patient and creating the corresponding database.

The variation in X-ray room layouts could be a potential challenge that was partly addressed by implementing the region-of-interest cropping strategy. This could be further enhanced by increasing the database with acquisitions from more facilities or by generating synthetic data with different backgrounds [30].

The runtime and performance of our prototype demonstrate the feasibility of our method for real-time use, enabling the reduction of exposure errors and unnecessary radiation dose delivered to patients due to retakes. The implementation of our method in a real system only requires a camera, an executable installed on the control PC, and a new GUI workflow which automatically selects the positions to be confirmed by the X-ray technician. Since radiography is the most common medical imaging modality used in the clinics [31], the incorporation of this method in radiological systems can significantly impact the healthcare system in terms of patient safety and radiology department workflow optimization.
