# Correction: Deep Learning–Based Estimation of Radiographic Position to Automatically Set Up the X-Ray Prime Factors

**DOI:** 10.1007/s10278-025-01476-9

**Published:** 2025-04-24

**Authors:** C. F. Del Cerro, R. C. Gimenez, J. Garcia-Blas, K. Sosenko, J. M. Ortega, M. Desco, M. Abella

**Affiliations:** 1 Bioengineering Department, Universidad Carlos III de Madrid, Leganés, Madrid, Spain; 2 Instituto de Investigación Sanitaria Gregorio Marañón, Madrid, Spain; 3 Computer Science and Engineering Department, Universidad Carlos III de Madrid, Madrid, Spain; 4 Sociedad Española de Electromedicina y Calidad S.A. (SEDECAL), Madrid, Spain; 5 Centro Nacional de Investigaciones Cardiovasculares Carlos III (CNIC), Madrid, Spain; 6 Centro de Investigación Biomédica en Red de Salud Mental (CIBERSAM), Madrid, Spain

**Correction: Journal of Imaging Informatics in Medicine**

10.1007/s10278-024-01256-x

This paper has been updated with open access.

